# Effectiveness of interventions for prevention of common infections in people who use opioids: a protocol for a systematic review of systematic reviews

**DOI:** 10.1186/s13643-021-01852-w

**Published:** 2021-11-15

**Authors:** Irina Kudrina, Svetlana Puzhko, Kristian B. Filion, Genevieve Gore, Elena Paraskevopoulos, Sarah Windle, Marc O. Martel, Mark J. Eisenberg

**Affiliations:** 1 Department of Family Medicine, Faculty of Medicine and Health Sciences, McGill University, 5858 Chemin de la Côte-des-Neiges, Suite 300, Montreal, QC H3S 1Z1 Canada; 2 Department of Anesthesia, Faculty of Medicine and Health Sciences, McGill University, 1650 Cedar Ave., Montreal, QC H3G 1A4 Canada; 3 Department of Epidemiology, Biostatistics and Occupational Health, Faculty of Medicine and Health Sciences, McGill University, Purvis Hall, 1020 Pine Avenue West, Montreal, QC H3A 1A2 Canada; 4 Lady Davis Institute for Medical Research, Jewish General Hospital, 3755 Chemin de la Côte-Sainte-Catherine, Montreal, QC H3T 1E2 Canada; 5 Department of Medicine, Faculty of Medicine and Health Sciences, McGill University Health Center, McGill University, 1001 Decarie Boulevard, Suite D05-2212, Montreal, QC H4A3JI Canada; 6 Schulich Library of Physical Sciences, Life Sciences, and Engineering, McGill University, 3459 rue McTavish, Montreal, QC H3A OC9 Canada; 7 Queensway Carleton Hospital, 3045 Baseline Rd, Ottawa, ON K2H 8P4 Canada; 8 Faculty of Dentistry, McGill University, 2001 Avenue McGill College, Suite 500, Montreal, QC H3A 1G1 Canada; 9 Department of Medicine, Faculty of Medicine and Health Sciences, McGill University, 3605 de la Montagne, Montreal, Qc H3G 2M1 Canada; 10 Division of Cardiology, Jewish General Hospital, 3755 Chemin de la Côte-Sainte-Catherine, Montreal, QC H3T 1E2 Canada

**Keywords:** Overview of systematic reviews, Umbrella review, Opioids, Infections, Prevention, Interventions, Risk reduction, Harm reduction

## Abstract

**Background:**

The North American opioid crisis is driven by opioid-related mortality and morbidity, including opioid use-associated infections (OUAIs), resulting in a substantial burden for society. Users of legal and illegal opioids are at an increased risk of OUAIs compared to individuals not using opioids. As reported for hepatitis C virus (HCV), human immunodeficiency virus (HIV), bacterial, fungal, and other infections, OUAIs transmission and acquisition risks may be modifiable. Several systematic reviews (SRs) synthetized data regarding interventions to prevent infections in persons using drugs (e.g., opioid substitution therapy, needle and syringes exchange programs, psycho-social interventions); however, their conclusions varied. Therefore, SR of published SRs is needed to synthesize the highest level of evidence on the scope and effectiveness of interventions to prevent OUAIs in people using opioids legally or illegally.

**Methods:**

We will comprehensively search for SRs in the PubMed, Embase, PsycINFO, Cochrane Database of Systematic Reviews, Epistemonikos, and Google Scholar databases from inception to November 2020. Data selection and extraction for each SR will be performed independently by two researchers, with disagreements resolved by consensus. All SRs regarding interventions with evaluated effectiveness to prevent OUAI in legal and/or illegal opioid users will be eligible. Risk of bias assessment will be performed using the AMSTAR2 tool. The results will be qualitatively synthesized, and a typology of interventions’ effectiveness with a statement on the strength of evidence for each category will be created.

**Discussion:**

Our pilot search of PubMed resulted in 379 SRs analyzing the effectiveness of interventions to prevent HCV and HIV in persons who inject different types of drugs, including opioids. Of these 379 SRs, 8 evaluated primary studies where participants used opioids and would therefore be eligible for inclusion. The search results thus justify the application of SR of SRs approach. Comprehensive data on the scope and effectiveness of existing interventions to prevent OUAIs will help policy-makers to plan and implement preventive interventions and will assist clinicians in the guidance for their patients using opioids.

**Systematic review registration:**

Registered in PROSPERO on 30 July 2020 (#195929).

**Supplementary Information:**

The online version contains supplementary material available at 10.1186/s13643-021-01852-w.

## Background

More than half a million lives have been lost to the North American opioid crisis [1–4]. While opioid-related mortality is staggering, opioid-associated morbidity often garners much less attention, despite its major health and economic burden to society [5, 6]. In particular, the prevention of acquisition and transmission of opioid use associated infections (OUAI) requires more attention by society and health care providers [7–10].

People who use illegally obtained pharmaceutical or non-pharmaceutical opioids [11], especially persons who inject drugs, are at high risk of serious infectious such as infective endocarditis, septic arthritis, osteomyelitis, meningitis, cellulitis, abscesses, and bacteremia, resulting in prolonged and expensive hospital stays [12, 13]. Hepatitis C (HCV) and human immunodeficiency virus (HIV) are two commonly cited OUAIs associated with a substantial burden for individuals and society: approximately 10–20% of HCV-infected individuals are at risk of hepatic cirrhosis, hepatocellular carcinoma, and hepatic failure in the 20–30 years post-viral acquisition [14]. Likewise, HIV infection causes a wide range of immunological problems, including infectious and oncological diseases, cardiovascular, bone, hepatic, and renal diseases, as well as chronic pain [15]. The risk of OUAIs, however, is not limited to illegal opioid consumption only. Patients treated with pharmaceutical opioids [11] appear more susceptible to viral, fungal, and bacterial infections when compared to patients not treated with opioids [16–21]. In higher doses, prolonged therapeutic use of opioids seems to facilitate virus entry and replication (hepatitis A, B, C, and HIV) and increases the risk of opportunistic bacterial, fungal, and parasitic infections [20, 21].

To prevent OUAIs in persons who inject drugs, the Centers for Disease Control and Prevention guideline [22] recommends screening for infectious complications and use of various preventive measures to reduce acquisition and transmission of HCV, HIV, hepatitis B virus, herpes simplex virus type 2, human papillomavirus, tuberculosis, and common sexually transmitted diseases. In addition, The Best Practice Recommendations for Canadian Harm Reduction Programs [23] highlight the importance of routine skin care to prevent bacterial and fungal infections in persons who inject drugs. For people who use opioids, additional risks for infections transmission and acquisition are related to opioids’ immunosuppressive properties. Thus, the risks are multi-factorial, and a variety of effective interventions aimed to prevent OUAIs are of paramount importance to the individuals, public, healthcare decision-makers, and policy advisers.

We, therefore, propose to conduct a systematic review (SR) of SRs (SR of SRs) to describe the scope and evaluate the effectiveness of the interventions to prevent OUAIs. The SR of SRs is a type of overview of reviews that are intended to inform guidelines and clinical practices [24] “…to provide…summaries of the breadth of research relevant to a decision without decision makers needing to assimilate the results of multiple systematic reviews themselves” [25–27].

To identify a potential scope of existing interventions to curb opioid crisis, an initial search was performed for an overarching research study (Canadian Institutes of Health Research [CIHR] grant #EOC-162067). Among others, this search revealed 18 SRs [28–45] on OUAIs prevention. The identified literature was limited to the HCV and HIV prevention, treatment, or integrated care, and targeted population of persons who inject drugs only. Therefore, some SRs devoted to other types of OUAIs prevention may have been missed.

Thus, the goal of the present SR of SRs is to synthesize SR-level evidence regarding interventions with evaluated effectiveness in prevention of OUAIs. We will include all categories of opioids use, legal, and illegal/mixed (see “Methods” and “Definitions” sections). All types of pharmaceutical and non-pharmaceutical opioids and all routes of use (injecting and non-injecting) will be eligible. Finally, for SRs studying the population of illegal/mixed opioid users, we will ensure that use or co-use of opioids was confirmed among study participants.

## Review goal and objectives

The overarching question for the SR of SRs in this protocol is: What is the SR-level evidence concerning the scope and effectiveness of interventions to prevent OUAIs?

The three specific objectives are (1) to describe SR-level of evidence for the scope of interventions with evaluated effectiveness in prevention of OUAIs; (2) to synthesize the SR-level evidence on the effectiveness of interventions to prevent OUAIs; and (3) to identify knowledge gaps in this area.

## Methods

### Study design

The proposed study design is based on the Systematic Review of Systematic Reviews (SR of SRs) [46] methodology, which is a subtype of the Overview of Systematic Reviews approach [26, 46]. Both SR of SRs and overviews of SRs are commonly called umbrella reviews as they synthetize review-based data. As compared to SR of SRs, overviews of SRs might not use a comprehensive set of information sources or an exhaustive systematic search strategy. On the other hand, SR of SRs methodology allows to perform a comprehensive review of the highest level of evidence [47, 48] from already synthesized SR-level data, with the end product ready to be used by clinicians and policy-makers. As multiple published SRs regarding existing interventions were identified in the preliminary grant search, we chose the SR of SRs methodology for this knowledge synthesis.

The protocol’s methodology is based on the approach described in the Cochrane Handbook for Systematic Reviews of Interventions, chapter 5 [26], Cochrane Handbook for Systematic Reviews of Interventions Version 510 [48], and on the Joanna Briggs Institute Reviewer’s Manual [49]. This SR of SRs will be reported according to the Preferred Reporting Items for Systematic Reviews and Meta-analyses (PRISMA) statement [50] and Preferred Reporting Items for Overview of Systematic Reviews (PRIO-harms) [46] (Please see PRISMA checklist, Additional file 2). This SR of SRs will synthesize the results of existing SRs on the topic of the effectiveness of the interventions to prevent OUAIs without re-synthesizing the results of primary studies.

### Main outcomes of the SR of SRs

The expected outcome for Objective 1 is the identification of the scope of interventions with evaluated effectiveness in prevention of OUAIs. All interventions (programs, policies, approaches etc.) with evaluated effectiveness and aimed to prevent OUAIs will be documented and relevant details will be described. The details will include the type of the intervention, where the intervention took place, population size, duration of the intervention, type of opioid use, and type of targeted infection. The expected outcome for the Objective 2 is a synthesis of the effectiveness of interventions aimed to prevent OUAIs. Measures of effectiveness of the intervention/program/policy will be effect measures of an association between infection/disease incidence (e.g., HCV seroconversion) and participation in the intervention, estimated by an odds ratio (OR), risk ratio (RR), or hazard ratio (HR). Since the unit of our overview is the SR, pooled effect measures will be reviewed; however, for SRs without meta-analyses, effect measures reported by original studies discussed in SRs will be considered. The expected outcome for Objective 3 will be the identification of knowledge gaps in the SR-level evidence on the effectiveness of the interventions to prevent OUAIs.

### Definitions

Opioid use will be defined as any opioid use via any route of administration, including opioid co-use with other substances. Pharmaceutical opioids are opioids manufactured by a pharmaceutical company and approved for medical purposes in humans [3] regardless of their legal/illegal origin. Legal use is the use of therapeutically prescribed pharmaceutical opioids [11] by the person to whom it was prescribed. Opioid use disorder [51], opioid misuse, and mixed use of pharmaceutical and/or non-pharmaceutical opioids will be categorized as illegal/mixed opioid use. People who use opioids both legally and illegally or use both pharmaceutical and non-pharmaceutical opioids will be defined as mixed users. OUAIs are infections acquired by a person actively using any opioid via any route. Co-infections are confirmed infections acquired by a person actively using opioids and already infected with another infectious agent. Preventive interventions are individual- or population-level programs, initiatives, or interventions designed to prevent infection transmission and/or acquisition in persons using legal or illegal opioids. Examples of such interventions include opioid substitution therapy and maintenance programs, needles and syringes exchange programs, safe heroin supply and safe injection site, distribution of bleach disinfectant, structured social and health support programs, and behavioral interventions.

### Search strategy/information sources

The following information sources will be searched:

1. *Databases*: we will systematically search PubMed, Ovid Embase, Ovid PsycINFO, Cochrane Database of Systematic Reviews, Epistemonikos, and Google Scholar databases from the inception date to November 2020. The search will be subsequently updated to include the most up to date data into the publication. We will use keywords and indexing terms for the following four concepts: opioids, infections, preventive interventions, and SRs. A medical liaison librarian was engaged in designing the search strategy (Additional file 1).
2. *Lists of references of eligible SRs* revealed by the database search will be screened for additional potentially relevant SRs.
3. *Grey literature* will be searched using Google Scholar. To do this, we will enter keywords and will continue search for relevant articles until 10 pages of results have been reviewed or until the search does not reveal any article not captured by previous searches.

The search strategy is based on the following four concepts joined by the Boolean operator “AND”: (1) opioids (all commonly used opioids, including generic and brand names, and synonyms); (2) infections (all common viral, bacterial, and other infections associated with opioid use); (3) preventive interventions (see “Methods” and “Definitions” sections); and (4) systematic reviews with or without meta-analysis. The example of search strategy, representing a complete search in PubMed through Ovid, is presented in the Additional file 1. The search strategy will be adjusted for each database as appropriate.

### Eligibility criteria

Included studies will be SRs synthesizing data regarding interventions to reduce or prevent infection transmission or acquisition among people who use opioids and reporting on the effectiveness of these interventions, with the use of narrative synthesis and/or meta-analysis. The following inclusion/exclusion criteria will be applied.

#### Participants/populations

Use of either pharmaceutical or non-pharmaceutical opioids and all routes of use will be included. SRs evaluating only pediatric populations, including teenagers, will be excluded. SRs with populations of opioid users using rare and unusual/experimental substances/experimental ligands or non-opium poppy plants (e.g., kratom) will be excluded.

#### Intervention(s), comparator group

We will include SRs on any policy, program, or other intervention that evaluated its effectiveness at targeting the prevention of OUAIs transmission or acquisition, including transmission and acquisition of co-infections. Infectious complications in people who use opioids could be related to opioids’ immunosuppressive properties or risky behaviours, which are more pronounced in already vulnerable populations (e.g., already infected opioid users, intensive care unit patients, elderly, immunosuppressed). Therefore, existing interventions targeting prevention of infections could also include immunization programs, behavioral interventions, and more. Only research where the preventive intervention was clearly identified and its effectiveness evaluated will be included. The SRs of interventions potentially non-relevant to developed countries (e.g., humanitarian aid for developing countries) will be excluded. The comparator group will be opioid users not receiving the intervention of interest or a population prior to implementation of the intervention.

#### Types of studies

Included studies will consist of SRs on interventions to prevent or reduce OUAIs transmission/acquisition among opioid users. SRs should include original studies, where an evaluation of effectiveness of these interventions was performed. A publication will be considered an SR if it (1) describes methods, including a systematic search strategy and inclusion/exclusion criteria; (2) performs a comprehensive search (using all relevant databases and an exhaustive search strategy); and (3) conducts a formal quality assessment of included studies using a validated tool. Data may be derived from any study type (e.g., experimental [randomized controlled trials, non-randomized trials, quasi-experimental studies] or observational). Only SRs with retrievable full-text articles will be included. SRs that used either a narrative synthesis and/or meta-analysis will be included. Eligible SRs will explicitly state that their primary studies included participants who used opioids; with no restrictions in terms of settings (e.g., inpatients, outpatients, epidemiological cohorts) or infectious diagnoses relevant to opioid use. We will exclude SRs where an intervention and/or its effectiveness measures were not clearly described. SRs, where specific outcomes are not specified, will not be included. Primary studies will not be reviewed. SRs published in languages other than English and French will be excluded.

### Data selection

The eligibility of publications will be assessed in a two-stage process. First, titles and abstracts of all citations identified through the search strategy will be screened by two members of the research team (IK and SP) independently. RAYYAN platform (available at McGill: https://libraryguides.mcgill.ca/rayyan#s-lg-box-13326907) will be used to record decisions and reasons for exclusion; any potentially relevant study will be carried forward. The retrieved eligible full-text articles will be exported using a reference manager software (Endnote X8.1) for duplicates removal and storage. Subsequently, full texts will be reviewed for eligibility independently by the same two researchers. Disagreements will be resolved by consensus through consideration or discussion; if necessary, another reviewer will be involved. The remaining publications will be included in the overview of SRs.

### Data management and reporting

Data will be extracted by two authors (SP and IK) independently using standardized pilot-tested data collection form. In accordance with Cochrane recommendations for overview of reviews [26], the following data will be extracted: basic information about systematic reviews (title, authors, year of publication, number of studies, and participants included in the systematic review), data about primary studies (title, authors, year of publication, study design, country of publication, funding source); SR’s search strategies (number and names of databases searched; date of last search update); systematic review’s population(s) (age, sex, ethnicity, exclusive use of opioids and/or co-use with other substances, routes of use, diagnosis [e.g., chronic pain, opioid use disorder, or both], type of opioid-use associated infection, relevant co-morbidities [as an indicator of possible immunosuppression], settings); interventions assessed in SRs (type and description); SR comparator population (where applicable), primary outcomes, SR limitations, and methodological quality/risk of bias. In case of unclear or missing data, authors will be contacted. In case authors do not respond, the gap in data coverage will be reported. Primary studies included within all identified SRs will be systematically explored for overlap using a data spreadsheet. Overlapping of primary studies between SRs will be handled by including the most methodologically rigorous and/or most recent SRs. In overlapping reviews, if any SR is found to contain some important information not included in other reviews, the review will be chosen for inclusion. Disagreements between the two reviewers will be resolved by consensus, with assistance of the third overview author when necessary. Extracted data will be recorded in an excel spreadsheet.

### Risk of bias (quality) assessment

Methodological quality/risk of bias of included SRs will be assessed using the AMSTAR 2 tool [52]. AMSTAR 2 is designed to assess quality of SRs that include studies of healthcare interventions (high, moderate, low, or critically low). The following critical domains will be appraised whether the SR protocol was registered before the start of review; appropriateness of the literature search; reasoning for study exclusion; appropriateness of meta-analysis [52]. In accordance with the AMSTAR 2 tool and the PRIO-harms (PRISMA-based) checklist for overview of SRs addressing health interventions [46], assessments of risk of bias for primary studies included in the SRs will be reported. The tools and methods (e.g., piloted forms, independently, in duplicate) used will be specified, as well as whether an evaluation of quality of evidence for outcomes of interest was performed. Tabular summaries of the assessments will be presented. Accounting for risk of bias when interpreting results and assessing the impact of publication bias will be appraised. While all eligible reviews will be included, the quality of data will be reflected when performing data synthesis. The quality assessment will be performed by two independent reviewers, disagreements will be resolved by discussion and consensus, with the help of a third reviewer when necessary.

### Strategy for data synthesis

In our SR of SRs, we will qualitatively synthesize the evidence from SRs on interventions to prevent OUAIs in opioid users and their effectiveness. Extracted data will be tabulated according to the types of interventions. In cases where overlapping SRs data are included, the extent of the primary study overlap will be mapped by providing a citation matrix, with a calculated corrected covered area [26, 46]. The number and size of overlapping primary studies and their contribution to the analysis [26, 46] will be narratively described. Qualitative analysis of findings by narrative synthesis will be performed. Narrative summaries of the outcome data contained within SRs will be presented with corresponding tables and resulting data will be grouped by the type of intervention. Included SRs will be mapped to a specific taxonomy of interventions according to the type of opioid use. The level of confidence regarding the effectiveness of each category of interventions will be defined as “no SR-level evidence to either support or discount the effectiveness”, “insufficient evidence to either support or discount the effectiveness”, “tentative evidence to support effectiveness”, or “sufficient evidence to support the effectiveness” [26, 33].

The framework [53, 54] is based on the review’s quality, the authors’ conclusions, and design and findings of the original studies (Additional file 3, Table 1).

Legal and illegal opioid user populations will be assessed separately as they are likely to have different risk factors and may require preventive measures tailored to the group. Based on the study findings, recommendations regarding interventions targeted to reduce/prevent infections in North Americans using opioids will be formulated. Once SR of SRs findings are available, our team will consult with our principal knowledge users and other key stakeholders to obtain additional insights regarding the interpretation of our findings, the identification of knowledge gaps, and further research directions. This process will allow for the implementation of better-informed practices and policies to prevent OUAI in persons using opioids.

## Discussion

The initial search conducted for the larger, overarching study (described in the “Background” section) revealed 18 SRs that evaluated risk reduction interventions of HCV/HIV acquisition in persons injecting drugs. We therefore expected most papers to be related to HCV and HIV infections in the population of persons who inject drugs, including opioids. Our pilot search conducted for this SR of SRs in the PubMed database produced 379 papers, of which 12 research papers were identified by both searches. Of these 12 studies, 8 were SRs discussing preventive interventions in the previously non-infected opioid users. Our preliminary full-text screening showed that six of these SRs would be eligible for inclusion in our SR of SRs. Two more SRs, found in PubMed and not captured by the initial search, would also be eligible for inclusion, thus resulting in a minimum of eight eligible SRs from the PubMed database only. Therefore, the results of our pilot search in PubMed justify the application of SR of SRs methodology.

Since the conclusions on intervention effectiveness varied for different SRs, this SR of SRs will help synthesize the highest level of evidence and understand the level of confidence regarding the effectiveness of discussed interventions. The results will be disseminated among knowledge users (patients, clinicians, opioid crisis response partners, professional societies, and Health Canada) and promoted to be included in the future guidelines. Our search strategy was designed to capture all types of infections in legal and illegal users of pharmaceutical and non-pharmaceutical opioids. Therefore, our SR of SRs will demonstrate if published SRs cover well this broader issue, or whether there are knowledge gaps that need to be addressed by the future research to assist policy-related decision-making in the context of the North American opioid crisis.

## Strength and limitations

To our knowledge, this will be the first SR of SRs that will synthesize SR-level evidence on the effectiveness of interventions for prevention of infections in people who use pharmaceutical and non-pharmaceutical opioids, legally, and/or illegally, via any route of administration. It will be conducted as a part of the effort to curb North American opioid crisis. The main strength of the present work will be its SR of SRs design as it allows for the provision of the highest level of existing evidence in an accessible form to assist knowledge users with the relevant information and will allow policy-makers to make informed decisions.

There are, however, some potential limitations. More specifically, as with any SR of SRs, this study is limited to the evidence stemming from the published SRs only, thus excluding the evidence from other types of literature. Moreover, although our definition of SR complies with the PRISMA checklist, it is possible that some potentially relevant publications will be excluded. However, this approach will allow us to exclude studies with possible flaws and evidence that may be biased. Furthermore, only SRs with original studies with evaluated effectiveness of interventions will be included, which will restrict the scope of discussed interventions. This will be done to synthesize the evidence on interventions with evaluated effectiveness to inform the stakeholders. In addition, publications in languages other than English and French will not be included. This limitation may cause language bias; however, our goal is to synthesized evidence on the interventions that can be considered in the North American context. The scarcity of randomized controlled trials on the topic of our research may be another limitation of the study; however, this problem is related to the nature of our review question: planning randomized trials on the preventive interventions with a control group may be unethical. Publication bias is also possible since studies that evaluated effectiveness as low may be less likely to be published. One of the limitations was categorization of opioid use into legal and illegal: unless a person was using pharmaceutical opioids as prescribed for therapeutic purposes, type of use was ultimately challenging to classify. Finally, the GRADE system for SR of SRs has not yet been developed; however, we will apply the method that has been previously applied to the published overview of reviews to evaluate the level of certainty of the evidence.

## Conclusions

Since the beginning of opioid crisis, the number of emergency care visits and hospitalizations in opioid users has been rising. One of the main reasons to seek medical help is OUAIs and their complications, especially in patients who inject opioids. Such infections as HCV and HIV can bring irreparable harm to patients’ health. What is more, endocarditis, bone, joint, skin, and soft tissue infections are difficult to treat, they require lengthy hospital stays (administration of parenteral antibiotics along with the surgery and/or wound care) and substantial outpatient resources, including physical rehabilitation. Other serious infections (e.g., pneumonia) may not be related to the route of use but are rather associated with the ability of opioids to supress immune system. They, likewise, may require difficult and prolonged treatment and rehabilitation courses. In many cases, the transmission and acquisition of OUAIs may be preventable. Our SR of SRs aims to synthesize the existing evidence on the effectiveness of interventions to prevent OUAIs and reveal knowledge gaps related to this topic. The results of the initial feasibility search suggest that the focus of SRs in this area has been prevention of HCV and HIV infections in the population of persons who inject drugs, and the data in other areas might be scarce or non-existent, especially regarding the synthesized evidence on non-HCV and HIV OUAIs as well as OUAIs in the population of legal opioid users and in opioid non-injectors. Opioids are a major North American public health problem, and the importance of such knowledge cannot be overestimated. Our SR of SRs will offer the highest level of evidence (SR-level evidence) on the effectiveness of interventions and will inform knowledge users and policy-makers on the planning, organization, and use of the interventions to prevent OUAI and decrease morbidity and mortality in opioids users.

## Supplementary Information

**Additional file 1.** Example of search strategy (PubMed) developed with the involvement of health sciences librarian.

**Additional file 2.** PRISMA-P 2015 checklist.

**Additional file 3: Table 1.** Types of evidence statements and the level of evidence that was required to support each statement [54].

## Data Availability

Not applicable.

## Abbreviations

OUAIs: Opioid use-associated infections
SR: Systematic review
HCV: Hepatitis C virus
HIV: Human immunodeficiency virus
