# Supplementary material for: Effectiveness of interventions for prevention of common infections in people who use opioids: a protocol for a systematic review of systematic reviews
Source: Syst Rev. 2021 Nov 15;10:298. doi: 10.1186/s13643-021-01852-w (PMC8591821; doi:10.1186/s13643-021-01852-w)
Supplement: Supplementary file 1 — Additional file 1. Example of search strategy (PubMed) developed with the involvement of health sciences librarian. [file 13643_2021_1852_MOESM1_ESM.docx]

**Additional file 1**

**Example of search strategy (PubMed)**

**developed with the involvement of health sciences librarian.**

Concept #1, Opioids:

("analgesics, opioid"[mh] OR "analgesics, opioid"[pa] OR "Controlled Substances" [majr] OR narcotics[mh:noexp] OR "Opioid-Related Disorders" [mh] OR narcotic*[tw] OR opiate*[tw] OR opioid*[tw] OR acetylmethadol[tw] OR alfentanil[tw] OR anileridine[tw] OR Benzomorphan*[tw] OR bezitramide[tw] OR buprenorphine[tw] OR butorphanol[tw] OR Codeine[tw] OR Dextromethorphan[tw] OR Dextromoramide[tw] OR Dextropropoxyphene[tw] OR dezocine[tw] OR Diamorphine[tw] OR dihydrocodein*[tw] OR Diphenylpropylamine[tw] OR Ethylmorphine[tw] OR Fentanyl*[tw] OR Heroin[tw] OR Hydrocodon*[tw] OR Hydromorphon*[tw] OR ketobemidone[tw] OR levacetylmethadol[tw] OR Meperidine[tw] OR Meptazinol[tw] OR methadone[tw] OR Morphan*[tw] OR Morphin*[tw] OR nalbuphine[tw] OR nicomorphine[tw] OR normethadone[tw] OR Opium[tw] OR Oripavine[tw] OR Oxycodone[tw] OR Oxymorphone[tw] OR Papaveretum[tw] OR Pentazocine[tw] OR pethidin*[tw] OR Phenazocine[tw] OR Phenoperidine[tw] OR phentanyl[tw] OR Phenylpiperidine[tw] OR Piritramide[tw] OR remifentanil[tw] OR Sufentanil[tw] OR sulfentanil[tw] OR sulfentanyl[tw] OR tapentadol[tw] OR Tilidine[tw] OR Tramadol*[tw] OR Actiq[tw] OR adolonta[tw] OR Amadol[tw] OR Anpec[tw] OR Ardinex[tw] OR Asimadolin*[tw] OR Astramorph[tw] OR Avinza[tw] OR Biodalgic[tw] OR Pethidine[tw] OR Carfentanil[tw] OR Codinovo[tw] OR Contramal[tw] OR Demerol[tw] OR Dicodid[tw] OR dihydrohydroxycodeinone[tw] OR dihydromorphinone[tw] OR Dihydrone[tw] OR Dilaudid[tw] OR Dinarkon[tw] OR Dolantin[tw] OR Dolargan[tw] OR Dolcontral[tw] OR Dolosal[tw] OR Dolsin[tw] OR Durogesic[tw] OR Duromorph[tw] or Epimorph[tw] OR Eucodal[tw] OR Exalgo[tw] OR Fentanest[tw] OR Fentora[tw] OR Fortral[tw] OR Hycodan[tw] OR Hycon[tw] OR hydrocodone bitartrate[tw] OR hydroxyacetanilide[tw] OR hydroxycodeinone[tw] OR Isocodeine[tw] OR Isonipecain[tw] OR Jutadol[tw] OR Kadian[tw] OR Dromoran[tw] OR Laudacon[tw] OR Levodroman[tw] OR Levodromoran[tw] OR Levo-dromoran[tw] OR Levorphan[tw] OR Lexir[tw] OR Lidol[tw] OR Lorcet[tw] OR Lortab[tw] OR Lydol[tw] OR Morphia[tw] OR Morphium[tw] OR MS Contin[tw] OR N Methylmorphine[tw] OR Nobligan[tw] OR Norco[tw] OR Numorphan[tw] OR Operidine[tw] OR opiate[tw] OR opioid*[tw] OR Opso[tw] OR Oramorph SR[tw] OR Oripavine[tw] OR Oxecta[tw] OR Oxiconum[tw] OR Oxycodeinon[tw] OR Oxycone[tw] OR Oxycontin[tw] OR Palladone[tw] OR Pancodine[tw] OR Percocet[tw] OR Pethidine[tw] OR Prontofort[tw] OR Propoxyphene[tw] OR Robidone[tw] OR Roxicet[tw] OR Roxicodone[tw] OR Skenan[tw] OR Sublimaze[tw] OR Takadol[tw] OR Talwin[tw] OR Thebaine[tw] OR Theocodin[tw] OR Theradol[tw] OR Tiral[tw] OR Topalgic[tw] OR Tradol[tw] OR Tradolpuren[tw] OR Tradonal[tw] OR Tralgiol[tw] OR Trama[tw] OR Tramadin[tw] OR Tramadoc[tw] OR Trama-Dorsch[tw] OR Tramadura[tw] OR Tramagetic[tw] OR Tramagit[tw] OR Tramake[tw] OR Tramal[tw] OR Tramex[tw] OR Tramundin[tw] OR Trasedal[tw] OR Ultram[tw] OR Vicodin[tw] OR Zamudol[tw] OR Zohydro[tw] OR Zumalgic[tw] OR Zydol[tw] OR Zytram[tw] OR people who inject drug*[tw] OR persons who inject drug*)

AND

Concept #2, SR:

(systematic review [ti] OR meta-analysis [pt] OR meta-analysis [ti] OR systematic literature review [ti] OR this systematic review [tw] OR pooling project [tw] OR (systematic review [tiab] AND review [pt]) OR meta synthesis [ti] OR meta-analy*[ti] OR integrative review [tw] OR integrative research review [tw] OR rapid review [tw] OR umbrella review [tw] OR consensus development conference [pt] OR practice guideline [pt] OR drug class reviews [ti] OR cochrane database syst rev [ta] OR acp journal club [ta] OR health technol assess [ta] OR evid rep technol assess summ [ta] OR jbi database system rev implement rep [ta]) OR (clinical guideline [tw] AND management [tw]) OR ((evidence based[ti] OR evidence-based medicine [mh] OR best practice* [ti] OR evidence synthesis [tiab]) AND (review [pt] OR diseases category[mh] OR behavior and behavior mechanisms [mh] OR therapeutics [mh] OR evaluation study[pt] OR validation study[pt] OR guideline [pt] OR pmcbook)) OR ((systematic [tw] OR systematically [tw] OR critical [tiab] OR (study selection [tw]) OR (predetermined [tw] OR inclusion [tw] AND criteri* [tw]) OR exclusion criteri* [tw] OR main outcome measures [tw] OR standard of care [tw] OR standards of care [tw]) AND (survey [tiab] OR surveys [tiab] OR overview* [tw] OR review [tiab] OR reviews [tiab] OR search* [tw] OR handsearch [tw] OR analysis [ti] OR critique [tiab] OR appraisal [tw] OR (reduction [tw] AND (risk [mh] OR risk [tw]) AND (death OR recurrence))) AND (literature [tiab] OR articles [tiab] OR publications [tiab] OR publication [tiab] OR bibliography [tiab] OR bibliographies [tiab] OR published [tiab] OR pooled data [tw] OR unpublished [tw] OR citation [tw] OR citations [tw] OR database [tiab] OR internet [tiab] OR textbooks [tiab] OR references [tw] OR scales [tw] OR papers [tw] OR datasets [tw] OR trials [tiab] OR meta-analy* [tw] OR (clinical [tiab] AND studies [tiab]) OR treatment outcome [mh] OR treatment outcome [tw] OR pmcbook)) NOT(letter [pt] OR newspaper article [pt]) OR "health technology assessment winchester, england"[Journal] OR "Evid Rep Technol Assess (Full Rep)"[Journal] OR "Evid Rep Technol Assess (Summ)"[Journal] OR "Int J Technol Assess Health Care"[Journal] OR "GMS Health Technol Assess"[Journal] OR "Health Technol Assess (Rockv)"[Journal] OR "Health Technol Assess Rep"[Journal]

AND

Concept #3, preventive interventions

("prevention and control" [Subheading] OR "Primary Prevention"[Mesh] OR "Centers for Disease Control and Prevention, U.S."[Mesh] OR "Early Medical Intervention"[Mesh] OR "Internet-Based Intervention "[Mesh] OR "Early Medical Intervention "[Mesh] OR "Early Intervention, Educational "[Mesh] OR "Preventive Health Services"[Mesh] OR "Needle-Exchange Programs"[Mesh] OR "Education"[Mesh] OR "National Health Programs"[Mesh] OR "Diagnostic Screening Programs"[Mesh] OR "Mandatory Programs"[Mesh] OR "Voluntary Programs"[Mesh] OR "Immunization Programs"[Mesh] OR "Regional Medical Programs"[Mesh] OR "Health Promotion"[Mesh] OR "Health Facilities, Proprietary"[Mesh] OR "Referral and Consultation"[Mesh] OR "Prescription Drug Monitoring Programs"[Mesh] OR "Preventive Health Services"[Mesh] OR "Preventive Medicine"[Mesh] OR "Primary Prevention"[Mesh] OR "prevention and control" [Subheading] OR "Public Health"[Mesh] OR "Consumer Health Information"[Mesh] OR "Health Promotion"[Mesh] OR "Community Mental Health Services"[Mesh] OR "Community Health Services"[Mesh] OR "Policy"[Mesh] OR "Public Policy"[Mesh] OR "Health Policy"[Mesh] OR "Drug and Narcotic Control"[Mesh] OR prevention[tw] OR primary prevention[tw] OR control[tw] OR intervention*[tw] OR preventive[tw] OR preventive program*[tw] OR prevention program*[tw] OR government program*[tw] OR policy[tw] OR policies[tw] OR health services[tw] OR screening[tw] OR immunization*[tw] OR health promotion[tw] OR prescription drug monitoring program*[tw] OR maintenance treatment[tw] OR "Opiate Substitution Treatment"[Mesh] OR substitution treatment[tw] OR substitution therapy[tw] OR needle exchange program*[tw] OR needle/syringe program*[tw] OR needle and syringe program*[tw] OR psychosocial intervention*[tw] OR risk-reduction intervention*[tw] OR risk reduction Intervention*[tw] OR behavioural intervention*[tw] OR Public Health Agency of Canada[tw])

AND

Concept #4, infections

(Infections[Mesh] OR Infect*[tw] OR communicable disease*[tw] OR virus disease*[tw] OR viral disease* OR viral illness*[tw] OR viral infection*[tw] OR bacterial infection*[tw])

Filters: Humans
