# Supplementary material for: Effectiveness of interventions for prevention of common infections in people who use opioids: a protocol for a systematic review of systematic reviews
Source: Syst Rev. 2021 Nov 15;10:298. doi: 10.1186/s13643-021-01852-w (PMC8591821; doi:10.1186/s13643-021-01852-w)
Supplement: Supplementary file 3 — Additional file 3: Table 1. Types of evidence statements and the level of evidence that was required to support each statement [54]. [file 13643_2021_1852_MOESM3_ESM.pdf]

## Additional file 3.

Table 1. Types of evidence statements and the level of evidence that was required to support each statement ( Palmateer et al., 2010)

Table 1 Types of evidence statements and the level of evidence that was required to support each statement.[a]

| *Evidence statement* | *Level of evidence* |
|---|---|
| Sufficient evidence from reviews to either support or discount the effectiveness of an intervention | Clear statement from one or more *core* reviews based on multiple robust studies, *or*<br>Consistent evidence across multiple robust studies within one or more *core* reviews, in the absence of a clear and consistent statement in the review(s) |
| Tentative evidence from reviews to either support or discount the effectiveness of an intervention | A tentative statement from one or more *core* reviews based on consistent evidence from a small number of robust studies or multiple weaker studies, *or*<br>Consistent evidence from a small number of robust studies or multiple weaker studies within one or more *core* reviews, in the absence of a clear and consistent statement in the review(s), *or*<br>Conflicting evidence from one or more *core* reviews, with the stronger evidence weighted towards one side (either supporting or discounting effectiveness) and a plausible reason for the conflict, *or*<br>Consistent evidence from multiple robust studies within one or more *supplementary* reviews, in the absence of a core review |
| Insufficient evidence from reviews to either support or discount the effectiveness of an intervention | A statement of insufficient evidence from a *core* review, *or*<br>Insufficient evidence to either support or discount the effectiveness of an intervention (either because there is too little evidence or the evidence is too weak), in the absence of a clear and consistent statement of evidence from (a) *core* review(s), *or*<br>Anything less than consistent evidence from multiple robust studies within one or more *supplementary* reviews |
| No evidence | No core or supplementary reviews of the topic identified, due possibly to a lack of primary studies |

[a]Modified from Ellis *et al.* 2003 [6].

References

Ellis, S., Barnett-Page, E., Morgan, A., Taylor, L., Walters, R., & Goodrich, J. (2003). HIV prevention: a review of reviews assessing the effectiveness of interventions to reduce the risk of sexual transmission: Health Development Agency London.

Palmateer, N., Kimber, J., Hickman, M., Hutchinson, S., Rhodes, T., & Goldberg, D. (2010). Evidence for the effectiveness of sterile injecting equipment provision in preventing hepatitis C and human immunodeficiency virus transmission among injecting drug users: a review of reviews. *Addiction, 105*(5), 844-859.
